# Promoter Motif Profiling and Binding Site Distribution Analysis of Transcription Factors Predict Auto- and Cross-Regulatory Mechanisms in *Arabidopsis* Flowering Genes

**DOI:** 10.3390/ijms262211152

**Published:** 2025-11-18

**Authors:** Eszter Virág, Beáta B. Tóth, Barbara Kutasy, Ágnes Nagy, Klaudia Pákozdi, József Péter Pallos, Gábor Kardos, Géza Hegedűs

**Affiliations:** 1 Department of Planetary Health, One Health Institute, Faculty of Health Science, University of Debrecen, Egyetem Sq. 1, 4032 Debrecen, Hungary; kg@med.unideb.hu; 2 Research Institute for Medicinal Plants and Herbs Ltd., Lupaszigeti Str. 4, 2011 Budakalász, Hungary; kutasybarbara@gmail.com (B.K.); aginagy.nagy@gmail.com (Á.N.); pakozdiklaudia94@gmail.com (K.P.); pallos.jp@gynki.hu (J.P.P.); 3 Department of Infection Control and Hospital Epidemiology, One Health Institute, Faculty of Health Science, University of Debrecen, Egyetem Sq. 1, 4032 Debrecen, Hungary; toth.beata@etk.unideb.hu; 4 Department of Plant Physiology and Plant Ecology, Institute of Agronomy, Georgikon Campus, Hungarian University of Agriculture and Life Sciences, Festetics Str. 7, 8360 Keszthely, Hungary; 5 Department of Information Technology and Its Applications, Faculty of Information Technology, University of Pannonia, Gasparich Márk Str. 18/A, 8900 Zalaegerszeg, Hungary; hegedus.geza@zek.uni-pannon.hu

**Keywords:** *Arabidopsis thaliana*, transcription factor binding, gene promoter, flowering

## Abstract

The transition to flowering in *Arabidopsis thaliana* is governed by complex transcriptional regulatory networks, in which promoter-associated *cis*-regulatory elements integrate both developmental and environmental cues. To investigate these regulatory interactions, we analyzed promoter motifs of 18 flowering-related genes using curated motif resources, including the Eukaryotic Promoter Database (EPD) and JASPAR, applying stringent statistical thresholds. Transcription factors (TFs), which were predicted to bind across all examined promoters, were designated as putative master regulators, resulting in the identification of 36 candidates, predominantly belonging to the MADS-box, DOF, and IDD families. Positional analyses revealed both proximal and distal binding sites, including a notable motif at −1024 in *PISTILLATA* and at +466 in *SEPALLATA3*, potentially indicative of autoregulatory control. Comparative analysis further identified 96 gene-specific associations, reflecting a balance between shared and specialized regulatory mechanisms. Treatment with β-aminobutyric acid (BABA), which has a flowering delaying effect, repressed SQUAMOSA and increased DOF-type TFs, indicating a chromatin-associated reprogramming process, which may coordinate the transcriptional suppression of flowering activators. These findings refine current models of floral regulatory networks and provide testable hypotheses regarding autoregulatory and cross-regulatory circuits in the control of flower development.

## 1. Introduction

The transition from vegetative growth to flowering in *Arabidopsis thaliana* is governed by transcriptional networks that integrate diverse endogenous and environmental inputs. Core regulators such as FLOWERING LOCUS T (FT), LEAFY (LFY), APETALA1 (AP1), and SUPPRESSOR OF CONSTANS OVEREXPRESSION1 (SOC1/AGL20), together with numerous MADS-box transcription factors (TFs), coordinate meristem identity and floral organ specification by activating or repressing downstream targets in response to photoperiod, temperature, and hormonal signals [1]. Within this framework, regulatory genes can be broadly distinguished by their hierarchical roles in transmitting and executing flowering signals. TF genes act as primary regulatory determinants, whereas mediator genes such as *FT*, *SOC1*, and *LFY* function as integrative hubs that channel inputs from multiple pathways, ultimately activating MADS-box structural genes including *AP1*, *AP3*, and *PISTILLATA* (*PI*) to establish floral organ identity [2,3,4].

Functional diversification among flowering regulators is mediated in part by their ability to form higher-order protein complexes. For example, MADS-domain TFs encoded by *SEPALLATA* (*SEP2/AGL4*), *AGAMOUS* (*AG*), *AP1*, and *PI* assemble into multimeric DNA-binding complexes that interact cooperatively with promoter sequences, ensuring precise spatiotemporal control of target gene expression. Such complexes include the well-characterized AP3–PI heterodimer, which associates with SEP proteins to form tetrameric “floral quartets”, a molecular basis for floral organ identity [5,6]. These assemblies enhance developmental robustness by reducing stochastic variation and conferring regulatory specificity within complex expression programs.

Despite extensive progress in dissecting the roles of individual regulators, the architecture of their promoter regions—particularly the spatial distribution, density, and positional specificity of transcription factor binding sites (TFBSs)—remains incompletely resolved. Promoter organization critically shapes transcriptional outcomes by determining the strength and selectivity of TF binding [7]. Proximal clusters of TFBSs near the transcription start site (TSS) are often indicative of direct regulatory activity, whereas distal TFBSs may contribute to chromatin-mediated enhancer–promoter interactions or combinatorial regulation [8,9,10,11]. Comparative analyses in peach and maize demonstrated that restricting motif discovery to a proximal window (−500 bp to +200 bp relative to the TSS) increased recovery of functional *cis*-elements and enhanced predictive power [10]. Reviews of plant *cis*-regulatory elements have further emphasized that core promoter clusters typically mediate transcription initiation directly, while distal motifs often contribute to tissue-specific or condition-dependent responses through looping interactions [11,12,13]. Thus, both motif density and positional context are critical parameters for understanding transcriptional hierarchies in flowering networks.

Computational approaches based on position weight matrices (PWMs) provide a systematic means to predict *cis*-regulatory motifs and TFBSs across promoter sequences [14]. When integrated with curated resources such as the Eukaryotic Promoter Database (EPD) and JASPAR, PWM-based analyses yield comprehensive maps of potential regulatory inputs, including TFs with broad binding potential that may function as master regulators [15,16,17,18]. Chen et al. [19] introduced the concept of master regulatory transcription factors (MRTFs) to describe TFs that act as central hubs within regulatory hierarchies, frequently controlling not only downstream developmental targets but also other TFs and associated microRNAs, thereby reinforcing feedback and feed-forward loops. Such mechanisms highlight the pivotal role of MRTFs in conferring robustness and specificity to floral gene regulatory networks. Importantly, assessing TFBS predictions under variable stringency thresholds can help distinguish robust interactions from context-dependent ones, thereby guiding experimental prioritization [20,21].

Building on these principles, the present study systematically analyzed the promoters of 18 flowering-related genes in *A. thaliana* to identify candidate MRTFs and to distinguish gene-specific from shared TFBS profiles (Table 1). The gene set included TFs (*SOC1/AGL20*, *LFY*, *FRUITFULL* (*FUL*)*/AGL8*, *SHORT VEGETATIVE PHASE* (*SVP*)*/AGL22*), mediator genes (*FT*, *TWIN SISTER OF FT* (*TSF*), *FLOWERING LOCUS D (FD*), and MADS-box structural genes (*AP1*, *AP3*, *PI*, *AG*, *SEP1/AGL2*, *SEP2/AGL4*, *SEP3/AGL9*, *SEP4/AGL3*). This functional diversity underscores the hierarchical nature of flowering control and provides a framework for investigating how TFBS composition contributes to both shared and gene-specific regulation. We hypothesized that TFBSs within these promoters would display distinct positional and quantitative patterns indicative of underlying regulatory complexity. Specifically, we asked whether (i) core flowering genes share a common set of TFs with binding potential across all promoters, (ii) particular TF families are preferentially enriched in proximal versus distal regions, and (iii) positional clustering reveals signatures of autoregulatory or cross-regulatory interactions. Through comprehensive PWM-based promoter scans and the compilation of predicted TFBSs for 489 TFs, this study provides a resource for modeling floral transcriptional networks and establishes a basis for future functional validation of autoregulatory and combinatorial regulatory modules. To support the theory of an integrated regulatory network of promoter motifs and TFs, transcriptomic responses to β-aminobutyric acid (BABA) treatment as an experimental validation and extended model within the context of epigenetic and stress signaling regulation were investigated. BABA has a strong influence on abscisic acid (ABA) (increase) and gibberellic acid (GA) (decrease) signaling, defense prioritization, and chromatin-mediated transcriptional control, leading to an overall delay in flowering and female sterility in *A. thaliana*. Therefore, BABA may act as a systemic modulator of both immunity and phenological plasticity [22].

## 2. Results

### 2.1. Distribution of Transcription Factor-Binding Sites in Flowering Regulatory Genes

To assess the regulatory complexity of flowering-associated genes, including TFs, mediator genes, and MADS-box structural genes, we analyzed the distribution of TFBSs within a genomic window spanning −5000 to +1000 bp relative to the TSS in 18 key flowering regulators, using promoter sequences from the EPD. Predicted TFBSs were identified under two statistical thresholds (*p* < 0.0001 and *p* < 0.00001), and their spatial distribution across upstream and downstream regions is summarized in Table 2.

At the *p* < 0.0001 threshold, upstream regions consistently contained more TFBSs than downstream regions across all genes. The highest upstream densities were observed in *PI* (855 sites), *SEP3* (788 sites), and *AP2* (752 sites), whereas *LFY* exhibited the lowest upstream count (502 sites). Interestingly, *LFY* displayed the highest number of downstream TFBSs (345 sites), suggesting a distinct regulatory architecture compared to other genes. By contrast, *PI* and *SEP1/AGL2* contained the fewest downstream sites (87 and 109, respectively).

At the more stringent *p* < 0.00001 level, the overall number of predicted TFBSs decreased, but upstream enrichment remained pronounced. The highest upstream counts were observed for *AP2* (202 sites), *SVP* (185 sites), and *SEP3* (181 sites), whereas *AGL24* and *SEP1* exhibited the lowest numbers (76 and 94, respectively). Consistent with the relaxed threshold, *LFY* retained an unusually high density of downstream TFBSs (105 sites), well above the average for other genes.

Overall, these findings confirm that TFBSs in flowering regulators are predominantly located in upstream promoter regions, consistent with canonical promoter organization. However, the enrichment of downstream TFBSs in *LFY* suggests the presence of non-canonical regulatory elements, potentially contributing to its complex role in floral induction and meristem identity.

In addition, 96 TFs were predicted to bind exclusively to a single gene among the 18 analyzed, representing gene-specific associations (Supplementary File S1). These were identified only under the lenient threshold (*p* < 0.0001). The highest numbers of gene-specific TFs were detected for *SOC1* (15 TFs), *FUL* (13 TFs), *SEP2* (12 TFs), and *PI* (11 TFs). Notably, *FUL* exhibited gene-specific sites predominantly in the downstream region, almost all belonging to the bHLH family (positions 640–641), except for one HD-Zip site (*HOMEOBOX ARABIDOPSIS THALIANA 2-HAT2*) at position 757. For *SOC1*, downstream-specific sites were enriched in WRKY family members (positions 626–633). By contrast, *PI* displayed extensive gene-specific activity in its upstream region (−1852 to −4693), with binding sites assigned to multiple TF families, including TCP, ERF, NAC, MYB, DREB, and AP2.

### 2.2. Identification of Putative MRTFs Regulating Flowering Genes in A. thaliana

**Figure 1:**
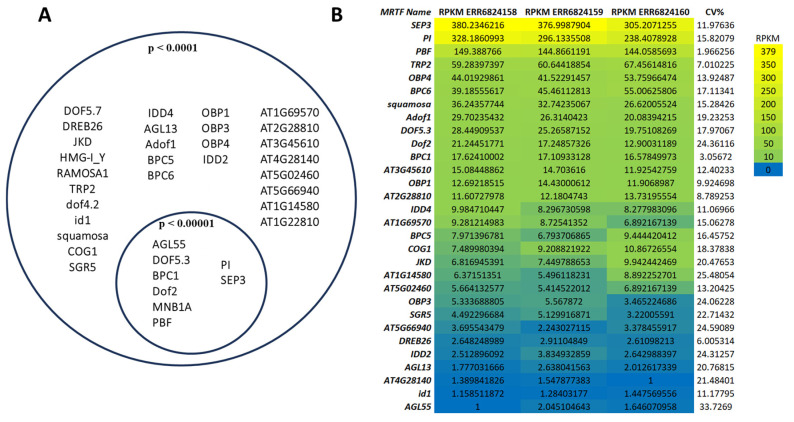
Overlap of identified MRTFs associated with the 18 flowering-time gene promoters (**A**). The left circle represents all predicted common MRTFs with the stringency *p* < 0.0001 binding in the regions −5000 to +1000 bp, and the right circle shows the subset of highly significant matches (*p* < 0.00001). The overlapping area contains the most confidently predicted core MRTFs. The identified MRTFs were investigated by their expression in floral tissues and visualized in a heatmap (**B**). To compare the expression of predicted MRTFs in the given sample, we calculated RPKM + 1 indexes. The genes are ranked from top to bottom based on their RPKM values. Warmer colors (yellow) indicate higher expression, while cooler colors (blue) represent lower transcript abundance. CV%: coefficient of variation.

To identify key transcriptional regulators of flowering-associated genes, including TFs, mediator genes, and MADS-box structural genes, we performed a comprehensive analysis of TFBS enrichment across the promoters of 18 core flowering regulators in *A. thaliana*. MRTFs were defined as TFs predicted to bind to all 18 promoter regions, regardless of binding site position. Using this criterion, we identified 36 candidate MRTFs, characterized by the frequent and statistically significant presence of binding sites within the upstream or downstream regions of all analyzed genes (Figure 1A).

The most robust candidates—those predicted to bind all 18 genes at both stringency levels—were located in the intersection of the Venn diagram (Figure 1A). These included AGL55, Dof5.3, BPC1, Dof2, MNB1A, PBF, PI, and SEP3. The overlap indicates that these TFs not only harbor motifs compatible with all investigated promoters but also do so with strong statistical confidence, suggesting that they may constitute a shared regulatory module. This finding is particularly consistent with the central role of MADS-box TFs in floral development.

Expression analysis of MRTFs in *A. thaliana* floral tissues (three biological replicates) revealed that 30 of the 36 MRTF genes were expressed (Figure 1B). Expression levels, ranked by mean Reads Per Kilobase per Million mapped reads (RPKM) values, varied substantially among candidates. *SEP3* and *PI* displayed the highest transcript abundances, consistently exceeding 300 and 200 RPKM, respectively. The presence of predicted binding sites within their own promoters supports the hypothesis that these genes may function not only as master regulators but also as autoregulatory components of the floral network.

In addition to MADS-box factors, TFs such as OBP1, OBP3, and OBP4 (OBF-binding proteins), along with IDD2, IDD4, and JKD (INDETERMINATE DOMAIN proteins), emerged as putative integrators of developmental cues and floral transition. Their known involvement in sugar signaling, meristem maintenance, and auxin pathways suggests broader roles in coordinating flowering with physiological states. By contrast, no expression was detected for HMG-I_Y, DOF4.2, DOF5.7, RAMOSA, AT1G14580, or MNB1A.

While the stringent threshold (*p* < 0.00001) reduces false positives, it may exclude biologically relevant low-affinity or context-dependent interactions. Therefore, to increase sensitivity and capture a broader spectrum of potential regulatory events, we adopted the more permissive threshold (*p* < 0.0001) for subsequent analyses.

### 2.3. Core MRTF Binading Sites Within the Proximal Promoter Region

Analysis of the central promoter regions between −500 and +200 bp, revealed that among the core MRTFs, BPC1 and OBP3 show a broad and conserved binding site distribution for the 18 genes studied (Table S1). In this analysis, positive binding site predictions for the core MRTFs were obtained only when applying the less stringent threshold of *p* < 0.0001, whereas the more stringent cutoff (*p* < 0.00001) yielded no detectable hits within the −500 to +200 bp promoter region. The majority of BPC1 motifs were located in close proximity to the TSS (−62, −50, −35, −14, −6, +2, +16, +18, +20, +25, +39, +40, +53, +57, +69, +94, +102, +110, +112 bp), with a particularly dense clustering in the region between −100 and +100 bp. This location suggests the direct regulation of transcription initiation and supports the central role of BPC1 in stabilizing the promoter architecture.

In contrast, OBP3 binding sites in the proximal region were more concentrated between −400 and −50 bp (e.g., −424, −417, −373, −338, −319, −297, −249, −223, −172, −136, −130, −102, −90, −67, −59 bp), supplemented by some positive positions after the TSS (+8, +20, +25, +35, +38, +55, +85, +107, +131, +147 bp). The distribution shows a dual pattern: an upstream dominance (−400/−100 bp), potentially indicating enhancer-like regulation, and a smaller presence downstream and directly near the TSS, suggesting fine-tuning of transcription initiation.

### 2.4. Direct Autoregulatory Binding Sites of PI and SEP3 TFs

In addition to their roles as master regulators of floral organ identity, both *PI* and *SEP3* contained predicted TFBSs within their own promoter regions, suggesting potential autoregulatory feedback mechanisms. For *PI*, six upstream binding sites were detected at *p* < 0.0001, located at −3851, −2567, −2495, −1287, −1024, and −971 bp. Among these, the site at −1024 also met the high-stringency threshold (*p* < 0.00001). For *SEP3*, nine upstream binding sites were identified between −2979 and +776 bp relative to the TSS, with the +466 site also supported at high stringency.

**Figure 2:**
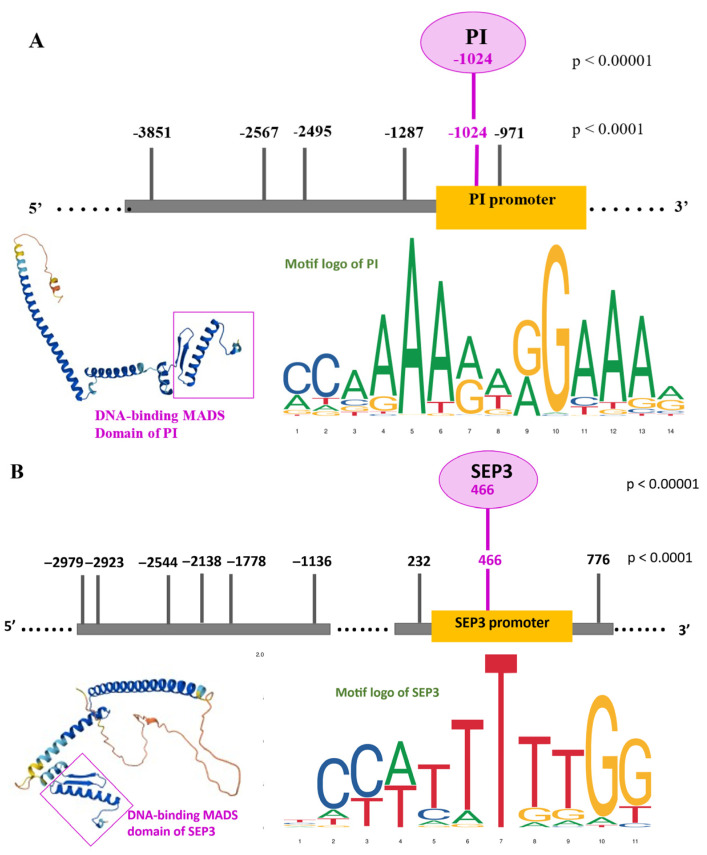
Direct autoregulatory binding sites of *PI* (**A**) and *SEP3* (**B**) TFs. The schematic figures of promoters indicate the found binding sites in the upstream positions of *PI* and both upstream and downstream regions of *SEP3* with both stringency thresholds. In the case of *PI*, in the upstream −1024 promoter position was found a direct BS with both stringencies, including the high *p* < 0.00001. In the case of *SEP3*, in the downstream +466 promoter position was found a direct BS with both stringencies, including the high *p* < 0.00001.

Both genes thus exhibited at least one high-confidence TFBS in close proximity to their own TSS. These positions, particularly −1024 in *PI* and +466 in *SEP3*, displayed strong sequence similarity to canonical MADS-box CArG motifs, reinforcing their potential biological relevance. The presence of such sites supports the hypothesis that *PI* and *SEP3* can directly bind to their own promoters, thereby participating in autoregulatory feedback loops (Figure 2A,B). In Figure 2 motif logos derived from genome-wide analyses indicate that *PI* and *SEP3* MADS-domain TFs preferentially bind to canonical CArG-box motifs (consensus: CC(A/T)_6_GG), often flanked by A/T-rich nucleotides. The high-confidence binding sites identified in the promoter regions of *PI* and *SEP3* are consistent with the characteristic base preferences revealed by the sequence logos. Sequence logos were downloaded from JASPAR database available on 3 August 2025 (https://jaspar.elixir.no/). The 3D structure (downloaded from AlphaFold database available on 3 August 2025 (https://alphafold.ebi.ac.uk/) of the DNA-binding MADS domain is shown, highlighting its helix-turn-helix-like architecture and the positively charged surface patches critical for DNA interaction. The inset reveals how the DNA-binding α-helices align with the major groove of the DNA, which corresponds spatially to the region where the CArG-box is situated in the *PI* and *SEP3* promoter.

### 2.5. Functional Analysis of Putative MRTFs

**Figure 3:**
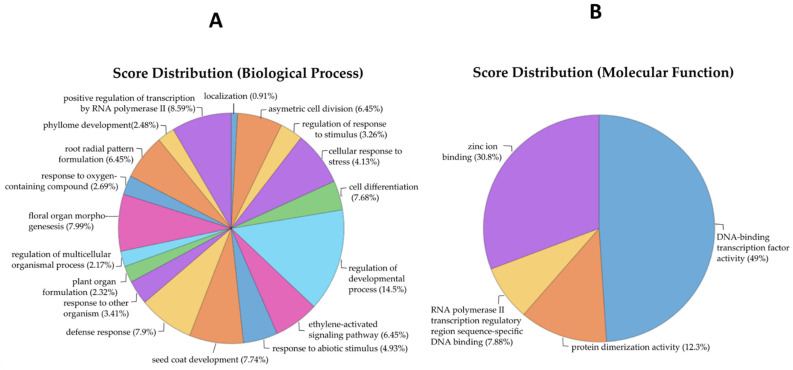
GO analysis of biological processes and molecular function linked to the identified MRTFs. Multi-level pie chart illustrating significantly enriched GO terms under the biological process (**A**) and molecular function (**B**) categories for the identified TFs associated with flowering in *A. thaliana*. The chart highlights the diverse regulatory functions of these TFs, including biological process categories such as regulation of developmental processes, defense response, cell differentiation, floral organ morphogenesis, and response to abiotic stimuli—underscoring their multifaceted roles in plant development, environmental adaptation, and stress signaling. The most prominent terms in molecular function categories include DNA-binding TF activity, zinc ion binding, protein dimerization activity, and RNA polymerase II transcription regulatory region sequence-specific DNA binding, reflecting the fundamental biochemical properties of these transcriptional regulators.

To investigate the functional roles of the identified MRTFs, a Gene Ontology (GO) enrichment analysis was conducted (Figure 3A,B; Supplementary File S2).

Within the biological process category, the most significantly enriched terms included regulation of developmental process (score: 6.728), floral organ morphogenesis (3.720), seed coat development (3.600), cell differentiation (3.576), and defense response (3.678). These terms are strongly associated with developmental transitions and reproductive phase regulation. Additional enrichment for response to abiotic stimulus (2.30), cellular response to stress (1.92), and response to other organisms (1.59) indicates a role in integrating environmental and endogenous signals. Terms such as ethylene-activated signaling pathway and root radial pattern formation (3.00 each) further suggest involvement of MRTFs in hormonal cross-talk and developmental plasticity.

For molecular function, strong enrichment was observed for DNA-binding TF activity (28.6) and zinc ion binding (18.0). Enrichment for protein dimerization activity (7.2) and RNA polymerase II transcription regulatory region sequence-specific DNA binding (4.6) supports the role of MRTFs as components of multiprotein complexes that directly regulate transcription initiation.

Cellular component analysis identified predominant localization to the nucleus (30.72), with a smaller contribution from the cytoplasm (5.936), consistent with their roles as transcriptional regulators.

Collectively, these findings reinforce the central function of MRTFs in controlling floral organ specification and developmental transitions, while also highlighting their broader involvement in stress signaling and hormonal regulation.

### 2.6. Pathway-Level Annotation of MRTFs Using Plant Reactome Database

**Figure 4:**
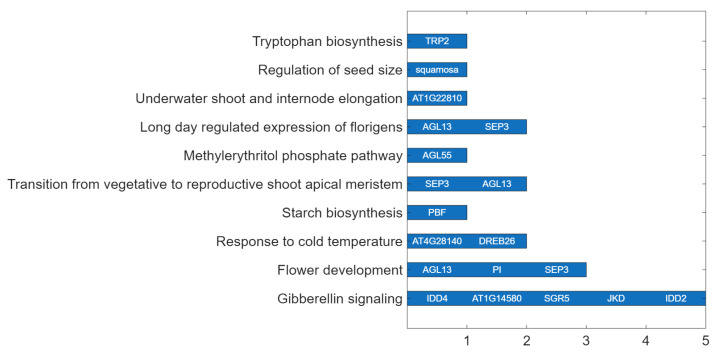
MRTFs associated with major Plant Reactome pathways regulating flowering and developmental transitions in *A. thaliana*. The bar chart displays the number of TFs identified for each pathway. Individual TFs are listed next to the corresponding bars. Notably, GA signaling, flower development, and the photoperiod-dependent floral induction pathways (e.g., long-day regulation of florigens, transition to reproductive shoot apical meristem) are regulated by multiple TFs, highlighting their central roles in flowering control.

To further clarify the biological context of the identified MRTFs, pathway enrichment analysis was performed using the Plant Reactome database (Figure 4).

Several MRTFs, including IDD4, SGR5, JKD, IDD2, and AT1G14580, were associated with the GA signaling pathway, highlighting their potential role in integrating hormonal cues with floral induction. TFs such as AGL13, PI, and SEP3 were linked to the flower development pathway, consistent with their established functions in floral organ identity and meristem determinacy. Moreover, SEP3 and AGL13 were annotated to both the vegetative-to-reproductive transition of the shoot apical meristem and the long-day–regulated expression of florigene, underscoring their importance in photoperiodic control of flowering.

In response to environmental cues, DREB26 and AT4G28140 were linked to the cold temperature response pathway, suggesting possible roles in cold-stress-induced flowering or in protective mechanisms during vegetative growth. Additional MRTFs were associated with specialized developmental or metabolic processes: PBF with starch biosynthesis, AGL55 with the methylerythritol phosphate (MEP) pathway (isoprenoid biosynthesis), and TRP2 with tryptophan biosynthesis, potentially influencing auxin production. Notably, SQUAMOSA was uniquely linked to the regulation of seed size pathway, while AT1G22810 was associated with underwater shoot and internode elongation, pointing to roles in developmental plasticity under stress or submergence.

Overall, these pathway-level associations reinforce the pleiotropic and integrative roles of MRTFs, linking flowering regulation with hormone signaling, stress adaptation, photoperiod responses, and key aspects of developmental metabolism.

### 2.7. Expression Profiling and Regulatory Roles of Floral MADS-Box Genes

**Figure 5:**
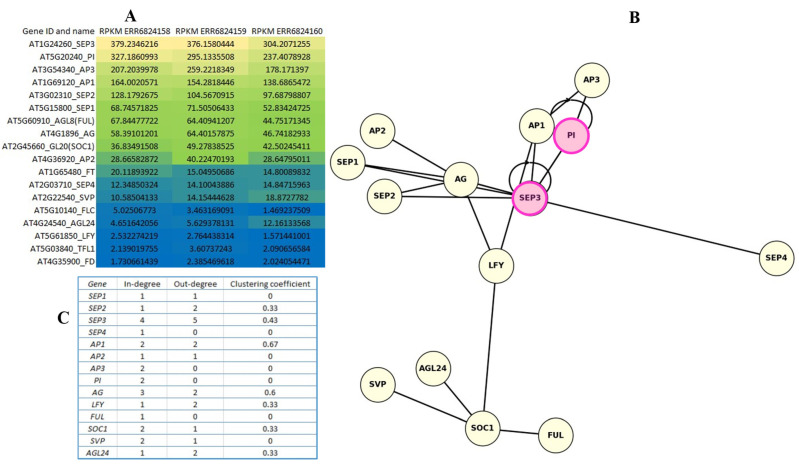
Expression heatmap of investigated genes including TF genes (*SOC1*, *LFY*, *FUL*, *SVP*), mediator genes (*FT*, *TSF*, *FD*), and MADS-box structural genes (*AP1*, *AP3*, *PI*, *AG*, *SEP1*, *SEP2*, *SEP3*, *SEP4*) (**A**). In the expression analysis, RPKM values were determined to compare the expression levels of genes within the sample. The heatmap uses a blue–green–yellow gradient to represent gene expression levels, where yellow indicates high expression, green medium expression, and blue low expression. The regulatory network illustrating the relationships of activation between key MADS-box and TF genes involved in *A. thaliana* flower development (**B**). Autoregulation is marked with pink circles: PI and SEP3 may activate themselves. PI and AP3 mutually activate each other (B-cluster dimer). Central actors are SEP3 and LFY: SEP3 also acts on several other SEP genes (SEP1, SEP2, SEP4) and AG, and vice versa. LFY is an activator of several important flower genes involving AP1, AG, SOC1, and may activate AGL24, SVP, SOC1, FUL, which are involved in flowering timing. The highly expressed genes PI and SEP3 act as regulatory hubs, where autoregulation and network node position can contribute to strong expression. The network topology values are indicated on the (**C**).

Expression patterns of the 18 flowering regulatory genes were analyzed in floral tissues using RNA-seq data, visualized as a heatmap of RPKM values (Figure 5A). Both *PI* and *SEP3* showed consistently high expression across all three replicates, ranking among the top four most abundantly expressed genes. Based on color gradient, genes were grouped into highly expressed (yellow), moderately expressed (green–blue), and low or non-expressed (dark blue). Several genes, including *LFY*, *FD*, and *TFL1*, exhibited minimal expression under the tested conditions.

In the regulatory network diagram (Figure 5B), *PI* and *SEP3* are highlighted in magenta, reflecting both their strong expression and evidence of autoregulation. *SEP3* exhibited the highest out-degree (5), suggesting regulation of multiple downstream targets, while *PI* appeared to act primarily through cooperative interactions, particularly with *AP3*. Notably, *SEP3* also displayed the highest in-degree (4), indicating regulation by several upstream TFs and integration of diverse signals.

The network topology (Figure 5C) illustrates the modular structure of the floral regulatory program, in which *AG*, *SEP3*, *AP1*, and *LFY* emerge as central hubs with multiple connections, consistent with their established roles in floral organ identity and developmental transitions.

### 2.8. Experimental Validation and Analysis of Epigenetic and Stress Signaling Regulation

Among the investigated 18 flowering-related genes, expression was detected in *SVP* and *SOC1* in vegetative samples. *SVP* showed consistent expression in vegetative tissues, with average RPM values of 23.9 in control and 31.4 in BABA-treated samples. The *AGL20* (*SOC1*) gene, a central activator of floral transition, exhibited low but detectable expression in vegetative tissues, with average RPM values of 4.16 and 4.98 in control and BABA-treated samples, respectively. The maintained and slightly increased *SVP* expression indicates that the vegetative repression module of flowering remained active under both conditions, supporting the persistence of a non-floral transcriptional state during BABA treatment. The slight increase in SOC1 under BABA treatment indicates that this gene remains transcriptionally repressed at the vegetative stage, consistent with the delayed floral induction observed in BABA-treated plants.

**Figure 6:**
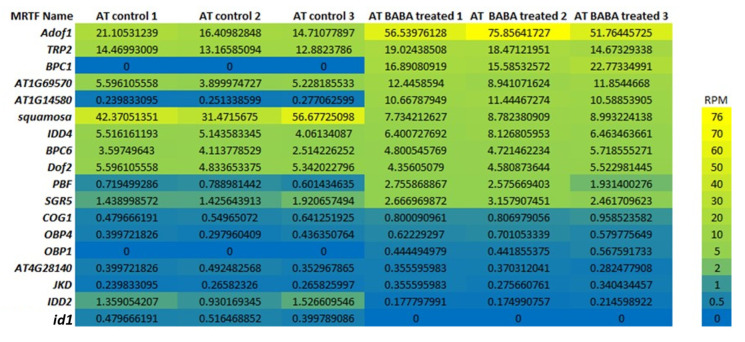
Expression heatmap. Transcriptomic responses of MRTFs to BABA treatment in AT vegetative tissues. Expression values are shown as RPM, indicating the normalized abundance of each transcript in RNA-seq libraries.

Transcriptomic responses of MRTFs to BABA treatment (Figure 6), as an experimental validation and extended model within the context of epigenetic and stress signaling regulation, indicated the following correlations: The marked decrease in *SQUAMOSA* expression under BABA treatment was detected (more than 5× decrease in RMP) This repression may contribute to the flowering delay by shifting transcriptional regulation toward stress-adaptive programs. The twofold increase in *AdoF1* and *AT1G69570* (*CDF5*) expression suggests enhanced activity of DOF-type TFs integrating metabolic and hormonal stress signals within the MRTF network. Furthermore, the exclusive expression of *BPC1* in BABA-treated plants may indicate the induction of chromatin-level regulatory mechanisms. The expression of *AT1G14580* (*AHL17*) markedly increased upon BABA treatment, suggesting the activation of a chromatin-level, epigenetic stress response. This gene encodes an AT-hook motif nuclear localized (AHL) family protein, a chromatin-binding transcriptional regulator that modulates gene expression through DNA structural changes. The induction of AHL17 suggests that BABA triggers chromatin-associated reprogramming linked to delayed flowering and simultaneously induces stress-response for defense in *A. thaliana.*

### 2.9. AlphaFold Modeling of PI and SEP3 Interactions with DNA

#### 2.9.1. Predicted DNA-Binding Interactions of PI and SEP3 Monomers

**Figure 7:**
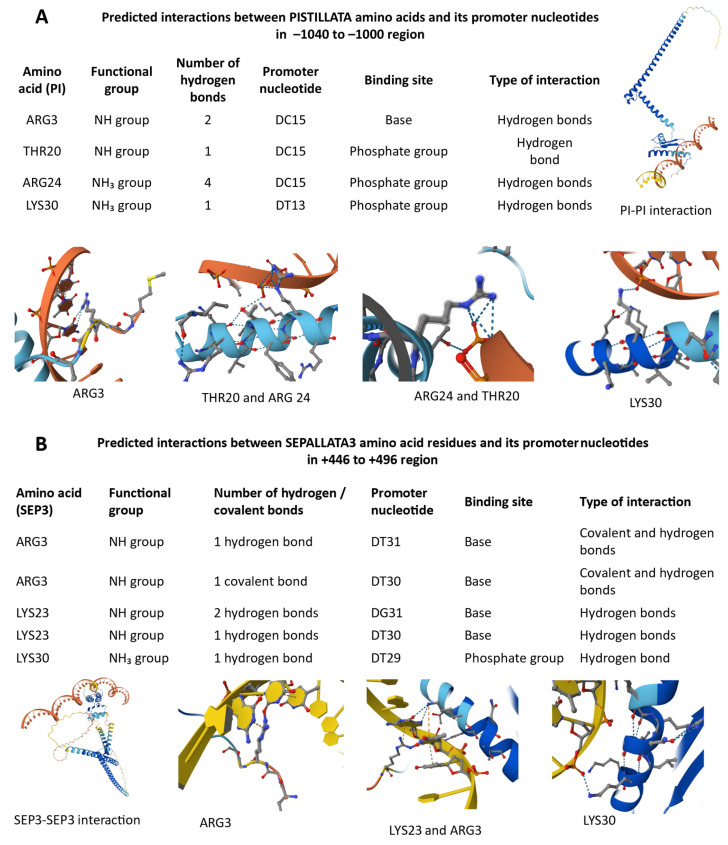
Predicted interactions between PI and SEP3 proteins and their respective promoter regions based on AlphaFold3 modeling. (**A**) *PI* monomer interactions within its promoter (–1040 to –1000 region). Hydrogen bonds are formed by PI residues ARG3, THR20, ARG24, and LYS30 with promoter nucleotides DC15 and DT13, indicating both base-specific and phosphate backbone contacts. (**B**) *SEP3* monomer binding to its own promoter (+446 to +496 region), showing covalent and hydrogen bond formation through ARG3, LYS23, and LYS30 with DT31, DG31, and DT29 nucleotides. Each panel illustrates amino acid–DNA contact sites and local secondary structure conformations of the interacting protein regions.

AlphaFold3 modeling predicted several potential interactions between PI amino acid residues and its own promoter region (−1040 to −1000). The analysis identified ARG3, THR20, ARG24, and LYS30 as key residues forming hydrogen bonds with the promoter nucleotides DC15 and DT13 (Figure 7A). ARG24 established the highest number of hydrogen bonds (four) with the phosphate group of DC15, suggesting a strong stabilizing contact with the DNA backbone. Both ARG3 and THR20 interacted with DC15, indicating a potential cooperative binding within this region. LYS30 also formed hydrogen bonds with the phosphate group of DT13, supporting its role in promoter recognition. In the −3870 to −3830 region of PI promoter, the residues ARG3, LYS23, ARG24, and LYS30 were found to interact with both the base and phosphate groups of promoter nucleotides DG25–DG27 and DT13 (Figure S1A). Among them, ARG24 exhibited the strongest interaction, forming three hydrogen bonds with the phosphate group of DG26, suggesting a central role in DNA binding stability. LYS23 showed versatile binding, interacting with multiple bases (DG25–DG27), while LYS30 and ARG3 contributed additional stabilizing hydrogen bonds to the phosphate backbone. The overall interaction pattern indicates that PI likely binds to its promoter through several basic residues, forming a network of hydrogen bonds indicating autoregulatory function in floral gene expression.

AlphaFold3 modeling predicted specific interactions between SEP3 amino acid residues and its own promoter region (+446 to +496) (Figure 7B). The residue ARG3 showed strong binding potential, forming both covalent and hydrogen bonds with DT30–DT31 bases, indicating a stable and potentially regulatory contact. LYS23 also contributed to promoter recognition by forming two hydrogen bonds with DG31 and DT30, while LYS30 established a single hydrogen bond with the phosphate group of DT29. These results suggest that SEP3 can self-associate and interact directly with its own promoter through multiple basic residues, particularly ARG3 and LYS23, which form complementary hydrogen- and covalent-bond networks. The observed SEP3–SEP3 interaction supports the model that SEP3 may autoregulate its transcription.

#### 2.9.2. PI–SEP3 Heterodimer Binding to the PI Promoter (−3870 to −3830 Region)

In the heterodimer model, both PI and SEP3 contributed to promoter recognition. The PI subunit residues LYS23, ARG24, and ARG3 participated in hydrogen bonding with DG26–DG27, while the SEP3 ARG3 residue formed multiple covalent and hydrogen bonds with DT20, indicating a stronger and more specific base interaction. These combined contacts may enhance the DNA-binding affinity of the dimer compared to the monomer, supporting cooperative regulation of the PI promoter by PI–SEP3 complexes (Figure S1B).

#### 2.9.3. PI–SEP3 Heterodimer Interaction with the PI Promoter (−1040 to −1000 Region)

At a downstream promoter region (−1040 to −1000), only SEP3 ARG3 was predicted to establish direct contact with DT18–DT19 nucleotides through multiple covalent bonds. However, no stable interaction was detected between the fully folded PI–SEP3 dimer and the DNA in this region, suggesting that local structural flexibility or cofactor recruitment may be required for effective binding in vivo (Figure S1C).

#### 2.9.4. PI–SEP3 Heterodimer Binding to the SEP3 Promoter (+446 to +496 Region)

In the SEP3 promoter, the PI component contributed to the interaction through ARG24 and LYS30 residues, each forming one hydrogen bond with phosphate groups of DG31 and DT29, respectively. Notably, ARG24 simultaneously interacted with the SEP3 GLU34 residue, suggesting an integrated DNA–protein–protein contact that stabilizes the heterodimer on the SEP3 promoter. These findings imply that PI may support SEP3 promoter recognition through electrostatic and cooperative interactions (Figure S1D).

## 3. Discussion

Although *A. thaliana* has been extensively investigated as a genetic model for flowering regulation, the present study provides a novel, integrative perspective on transcriptional control by focusing on promoter-level motif organization and TFBS architecture. By combining multi-threshold motif prediction, positional binding analysis, and pathway-level functional annotation, this work advances beyond previous studies centered mainly on gene expression or protein–protein interactions. The identification of thirty-six candidate MRTFs, together with the discovery of direct autoregulatory motifs in *PISTILLATA* and *SEPALLATA3*, reveals new dimensions of regulatory complexity. These results highlight the existence of interconnected auto- and cross-regulatory circuits that reinforce hierarchical control within the floral gene network of *A. thaliana*. Consequently, this approach not only complements but also extends established models of flowering regulation by elucidating the promoter-based mechanisms that underpin transcriptional hierarchy and developmental precision.

### 3.1. Spatial TFBS Distribution Reflects Gene-Specific Regulatory Modes

In this study, we performed a comprehensive in silico analysis of TFBS distributions across promoter regions of flowering-related genes in *A. thaliana*. By integrating PWM-based predictions with curated promoter annotations (EPD, JASPAR), we observed substantial gene-specific variation in both TFBS density and positioning. Notably, enrichment patterns were asymmetric: *PI*, *AP2*, and *SEP3* displayed high upstream TFBS density, whereas *LFY* showed pronounced downstream enrichment, indicative of distinct regulatory modes. These findings are consistent with genome-wide analyses in *A. thaliana*, which reported that ~86% of TFBSs are concentrated within −1000 to +200 bp relative to the TSS, with a peak ~50 bp upstream [23].

The detection of 96 gene-specific TFs at *p* < 0.0001 underscores the existence of highly specialized regulatory mechanisms. For example, *SOC1* and *FUL* exhibited downstream localization of gene-specific TFs, suggesting reliance on non-canonical regulatory elements [24]. Within *FUL*, bHLH motifs clustered near positions 640–641, while *SOC1* was targeted by WRKYs, potentially linking stress-responsive pathways with flowering control. In contrast, *PI* harbored diverse upstream gene-specific TFBSs (−1852 to −4693), spanning multiple TF families (e.g., TCP, MYB, NAC), indicative of complex multilayered regulation. Together, these patterns highlight the existence of distinct promoter architectures that reflect gene-specific regulatory strategies.

Genome-wide studies in *A. thaliana* have shown that TFBSs generally follow a bell-shaped distribution, peaking ~50 bp upstream of the TSS, with the majority localized within the −1000 to +200 bp window [23]. While this global pattern provides a broad reference, it does not capture the asymmetries identified here, such as the downstream bias of *LFY* or the upstream complexity of *PI*. Supporting this view, massively parallel reporter assays (MPRAs) demonstrated regulatory activity not only within canonical upstream regions (−40 to −200 bp) but also downstream of the TSS (+40 to +360 bp), confirming that functional TFBSs can extend into transcribed regions [25]. These observations suggest that promoter architecture in flowering regulators is non-uniform, with positional variability contributing to regulatory specificity and fine-tuning of gene expression.

Taken together, our findings provide a comparative framework for interpreting promoter complexity in flowering regulators. While the results are hypothesis-generating and should not be regarded as exhaustive, they emphasize that spatial TFBS distribution is a critical determinant of gene-specific regulatory modes.

### 3.2. Master Regulators Orchestrate Hierarchical Floral Control

The extensive TFBS landscapes of LFY and AP2, two well-established master regulators of floral identity, underscore their roles as central hubs that integrate diverse upstream signals. Dense TFBS clustering near the TSS likely reflects direct transcriptional activation, while distal and downstream elements may support chromatin remodeling, feedback regulation, or cross-pathway integration [26,27,28]. Indeed, LFY has been shown to bind 1588 genomic regions corresponding to 1296 genes at the seedling stage, with additional binding peaks emerging in inflorescence tissues. These binding sites are highly enriched near TSSs, reinforcing LFY’s role in promoter-proximal activation [29]. Re-analysis of ChIP-seq data for major floral regulators further revealed that LFY, SEP3, and BLR possess the most extensive binding repertoires, identifying them as key regulatory hubs. Such cross-regulatory and autoregulatory interactions are consistent with a hierarchical transcriptional framework governing floral development in *A. thaliana* [19]. Similarly, AP2 binds thousands of loci in developing floral tissues and engages in feedback regulation involving the miR156/miR172 axis, reinforcing its central position in floral gene networks [30].

Our identification of putative MRTFs—including AGL13, PI, SEP3, and Dof5.3 from the MADS-box, DOF, BPC, and IDD families—further supports the existence of a hierarchical regulatory structure in which MRTFs coordinate gene modules controlling meristem identity, organ specification, and developmental timing. This is consistent with large-scale network analyses in *A. thaliana*, where 11 major floral TFs (including AG, AP1, AP2, AP3/PI, SEP3, LFY, BLR, and FD) were found to regulate hundreds of targets [31,32]. Within these networks, LFY, SEP3, and AP2 emerge as particularly highly connected TFs, occupying top-tier positions in the regulatory hierarchy. The involvement of AGL13, PI, and other MADS-box family members, alongside AP2-class and DOF/IDD TFs, points to a modular yet integrated network architecture. Among these, SEP3 and AP2 stand out as central hubs, consistent with their known roles in floral organ identity.

To reduce potential ambiguity in associating distal TF binding sites, we additionally performed a core promoter analysis focusing on sites located within −500 to +200 bp around the TSS. The results showed the following: BPC1 and OBP3 adopt distinct but complementary regulatory strategies: BPC1 binding is strongly concentrated near the TSS, consistent with direct transcriptional activation, while OBP3 displays a broader upstream distribution suggestive of integrative, enhancer-like functions. This dual pattern aligns with models of master regulators as versatile hubs that combine proximal promoter control with distal regulatory interactions to integrate developmental and environmental signals [33,34].

### 3.3. Autoregulatory Potential, Expression and Network Relevance of PI and SEP3 TFs

The identification of high-confidence TFBSs within the promoter regions of *PI* and *SEP3* provides strong evidence for potential autoregulatory feedback in these key floral regulators. Autoregulation is a well-established strategy among MADS-box TFs to confer robustness, temporal precision, and spatial specificity during floral development [35,36].

For *PI*, six upstream TFBSs were predicted, including a site at −1024 bp relative to the TSS detected under both relaxed (*p* < 0.0001) and stringent (*p* < 0.00001) thresholds. This site is proximal enough to influence promoter activity and exhibits a strong match to the canonical CArG-box motif, underscoring its functional plausibility.

AlphaFold3 modeling revealed that both PI and SEP3 TFs can directly interact with their own promoters as well as with each other’s regulatory regions. The predicted hydrogen and covalent bonds between key positively charged residues (ARG and LYS) and promoter nucleotides suggest potential autoregulation and cooperative binding within floral gene networks. These interactions highlight a structural basis for the PI–SEP3 dimer’s role in fine-tuning floral organ identity gene expression [37].

To our knowledge, this represents the first in silico evidence of a high-confidence autoregulatory motif in *PI*. Such a finding expands the regulatory role of *PI* beyond its established function in B-function complexes through heterodimerization with *AP3* and interactions with *SEP* proteins [38,39,40].

For *SEP3*, nine upstream binding sites were detected, with the +466 bp site also passing the stringent threshold. Although autoregulation of *SEP3* has been previously hypothesized and partially supported experimentally [37,41,42], this specific site has not been highlighted before and may represent an additional functional element. The structural features of MADS-domain proteins further support this mechanism, as their DNA-binding domains are optimized for recognizing CArG motifs [43]. Alignment of sequence logos for PI and SEP3 with predicted sites confirmed high motif specificity, while structural models of the MADS domain indicated compatibility with DNA groove geometry and electrostatic properties, providing mechanistic plausibility [44,45].

Together, these predictions suggest that PI and SEP3 may employ autoregulatory feedback to reinforce expression stability and robustness within floral regulatory networks. Functional validation through promoter mutagenesis or ChIP-qPCR targeting the −1024 (*PI*) and +466 (*SEP3*) regions will be essential to confirm these hypotheses.

Consistent with this view, RNA-seq analysis revealed high expression of *PI* and *SEP3* across floral replicates [46]. Network analysis further placed both genes as regulatory hubs, with *SEP3* exhibiting the highest out-degree, suggesting broad influence over downstream targets [47]. By contrast, *PI* appears to function within cooperative modules, particularly through mutual activation with *AP3* in the B-function complex. These distinct strategies—broad connectivity for *SEP3* versus cooperative specialization for *PI*—likely reinforce their elevated transcript levels and centrality in the floral gene regulatory network.

GO and pathway analyses confirmed that the identified MRTFs are associated with core developmental, hormonal, and stress-responsive pathways, including GA signaling, floral organogenesis, and cold stress response. These findings emphasize the integrative roles of MRTFs in translating environmental stimuli into transcriptional reprogramming during the floral transition. In particular, the association of DREB26, IDD4, and SGR5 with abiotic stress and hormone-mediated processes illustrates the transcriptional plasticity that enables flowering to be finely tuned in response to both environmental and endogenous cues.

Members of the IDD family exemplify this regulatory flexibility. *SGR5* (*AtIDD15*) has been implicated in gravitropic response and auxin coordination, while *IDD4* regulates temperature and drought responses via ABA-mediated signaling [48]. These TFs therefore serve as integrators of environmental stress with hormone-regulated pathways. Similarly, transcriptome analyses show that DREB family members, including *DREB26*, are activated by cold and high-light stress and frequently co-cluster with TFs linked to auxin- and GA-mediated development [27]. This highlights extensive cross-talk between environmental and developmental pathways.

Systems-level transcriptomic studies under drought and other abiotic stresses further identify regulatory clusters containing *IDD4* and DREB factors, enriched for GO terms related to ABA and GA signaling, stress adaptation, and developmental control [49]. Together, these observations strongly support the view that MRTFs act at the interface of flowering regulation and environmental responsiveness, coordinating transcriptional reprogramming to ensure developmental plasticity.

### 3.4. Epigenetic and Stress-Induced Modulation by BABA Treatment

BABA has been shown to exert long-term regulatory effects through both signaling and epigenetic mechanisms, reprogramming transcriptional networks to enhance stress resilience while delaying developmental transitions such as flowering in *A. thaliana* [50,51]. The expression dynamics of flowering-related genes under BABA treatment indicate that the vegetative repression module remains active, while stress-responsive chromatin regulators are induced. The consistent expression and slight upregulation of *SVP* suggest maintenance of the SVP–FLC repression complex, which delays flowering by inhibiting SOC1 and FT [52,53,54]. The low *SOC1* expression, with only minor induction under BABA, confirms that floral activation remains suppressed, consistent with delayed flowering [55]. A strong repression of *SQUAMOSA*, a meristem identity gene [56], further indicates that BABA shifts transcriptional priorities from reproductive to defense programs. In contrast, the increased expression of *AdoF1* and *CDF5*, both DOF-type TFs, points to enhanced integration of metabolic and hormonal stress signals within the flowering network [57,58,59]. The exclusive induction of *BPC1* and the marked increase in *AHL17* (AT1G14580) expression under BABA treatment provide strong evidence for the activation of epigenetic and chromatin-level regulatory mechanisms. BPC proteins act as architectural TFs that mediate Polycomb group -dependent chromatin remodeling, influencing developmental gene silencing [60,61]. Similarly, *AHL17* encodes an AHL protein that binds to AT-rich DNA regions and modulates gene expression through local chromatin architecture changes. The upregulation of *AHL17* under BABA treatment suggests that stress signaling triggers a chromatin-associated reprogramming process, which may coordinate the transcriptional suppression of flowering activators [62]. These findings experimentally validate the role of the identified MRTFs in floral development under BABA treatment, which modulates MRTF activity to reinforce vegetative maintenance and establish a stress-primed, delayed-flowering phenotype in *A. thaliana.*

### 3.5. Methodological Insights from Multi-Threshold Motif Prediction

A key methodological outcome of this study is the value of applying multiple stringency thresholds in TFBS prediction, which enables separation of high-confidence interactions from context-dependent or conditionally relevant ones. By comparing *p*-value cutoffs (*p* < 0.0001 and *p* < 0.00001), we distinguished robust, high-affinity binding events from weaker predictions that may nevertheless contribute to regulation under specific developmental or environmental conditions.

This approach aligns with findings from a large-scale analysis of 111 *A. thaliana* ChIP-seq datasets, where binding site predictions were evaluated across three motif models (PWM, BaMM, SiteGA) and multiple recognition thresholds. That study showed that stringent cutoffs (false positive rate ≤ 1 × 10^−4^) predominantly identified high-affinity, evolutionarily conserved sites, consistent with conventional PWM-based predictions. In contrast, relaxed thresholds captured additional, weaker binding events often missed by PWM alone, likely reflecting context-specific or cooperative TF–DNA interactions [63].

Together, these observations highlight the interpretive power of multi-threshold analyses. Stringent criteria enrich conserved, high-confidence regulatory events, while relaxed thresholds broaden the search space to include dynamic or conditional interactions. Incorporating both perspectives refines candidate prioritization for experimental validation and facilitates reconstruction of regulatory hierarchies by emphasizing consistently predicted TF–target relationships.

### 3.6. Limitations of PWM-Based in Silico Predictions

Despite the insights gained, PWM-based predictions have inherent limitations. The assumption of nucleotide independence across motif positions can result in both false positives and false negatives, as shown in benchmarking studies where PWMs frequently failed to capture inter-nucleotide dependencies [64].

Moreover, TF binding in vivo is strongly influenced by epigenetic and structural features—including chromatin accessibility, DNA methylation, nucleosome positioning, and protein–protein interactions—that are not accounted for in sequence-based models. As a result, PWM analyses should be viewed primarily as hypothesis-generating tools, with predictions requiring careful experimental validation [65,66,67,68].

Future work should prioritize the experimental confirmation of high-confidence TF–target interactions through in planta approaches such as chromatin immunoprecipitation followed by qPCR (ChIP-qPCR), yeast one-hybrid assays, or promoter–reporter fusions in transgenic lines. In addition, the integration of genome-wide datasets—including DNA affinity purification sequencing (DAP-seq), ChIP-seq, and ATAC-seq—will be critical for refining TFBS maps and capturing context-dependent binding dynamics under diverse developmental and environmental conditions.

### 3.7. Outlook and Broader Applicability

This study provides a valuable resource for deciphering *cis*-regulatory mechanisms in *A. thaliana* and establishes a foundation for experimental validation of predicted MRTFs and their binding site interactions. By applying promoter motif profiling, we identified candidate autoregulatory and cross-regulatory elements in flowering genes, offering new hypotheses on the architecture of floral transcriptional networks.

Comparable genome-wide approaches have demonstrated similar utility in other contexts—for example, motif-based analyses of defense-related gene families, such as chitinases in apple, have uncovered lineage-specific expansions and stress-responsive regulation [69,70]. The workflow presented here can thus be readily extended to additional plant species, enabling the investigation of conserved and lineage-specific regulatory modules that shape floral development.

## 4. Materials and Methods

### 4.1. Gene Selection and Promoter Regions

Eighteen *A. thaliana* genes involved in floral meristem identity, flowering time regulation, and organ specification were selected based on their central role in regulatory pathways. GO names (BP, MF, CC) of these 18 selected genes are presented in Table 1. Promoter regions were defined according to EPD annotations, encompassing 5 kb upstream and 1 kb downstream of the TSS.

### 4.2. TF Binding Prediction

Literature comparisons of protein–DNA binding models indicate that PWMs perform comparably to more complex or computationally intensive methods in many contexts [71]. Therefore, we utilized the JASPAR TF motif library to obtain high-confidence PWMs for TFBS prediction, specifically JASPAR CORE 2018 plants [18]. The JASPAR TF motif library was accessed through its integration within the EPD interface (https://epd.epfl.ch (accessed on 1 March 2025)) to identify putative transcriptional regulatory elements within the promoter regions of the selected genes. JASPAR offers a comprehensive collection of TF binding motifs, including key core promoter elements such as the Initiator (Inr), TATA box, GC box, and CCAAT box. The JASPAR library includes PWM data for 489 TFs in *A. thaliana*. To minimize false-positive predictions during binding site evaluation, we applied two stringent *p*-value thresholds when using the EPD interface in this study. The results are hypothesis-generating and should be interpreted as a comparative overview rather than promoter-exclusive predictions.

**Figure 8:**
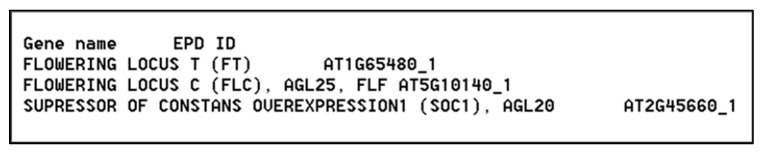
Structure of the ID file used for the Python script.

Regulatory sequences were analyzed across regions spanning 5 kb upstream and 1 kb downstream of the TSS, corresponding to the maximum allowable range supported by the EPD web service. To perform the TFBS queries, we used an in-house software tool, tfcollect v2.0, which is publicly available on GitHub at: https://github.com/DEpt-metagenom/tfcollectv2.0/blob/main/tfcollectv2.0.zip (accessed on 1 March 2025). To investigate differences in TFBS distributions between upstream (–) and downstream (+) promoter regions, we analyzed filtered values from the dataset using a custom Python script provided in Figure S2. The script processes input through an ID file formatted as a tab-delimited .txt file, containing gene names alongside their corresponding EPD identifiers. An example of the input file structure is shown in Figure 8.

### 4.3. Data Processing and Analysis

TFBS counts were extracted from both upstream and downstream regions and categorized by gene and by *p*-value threshold. The complete dataset has been deposited to Mendeley Data https://doi.org/10.17632/nzyw9csjs8.2 (accessed on 1 June 2025).

### 4.4. Functional Annotation and GO Term Analysis

To explore the potential biological roles of the identified MRTFs associated with flowering regulation in *A. thaliana*, a functional annotation and GO analysis was conducted using the OmicsBox platform [72] (BioBam Bioinformatics, Valencia, Spain). This analysis was based on a predefined set of putative master regulators rather than differentially expressed genes referring to the GO categories within the input list relative to a reference genome background and expression-based fold changes.

The enrichment scores reported correspond to the negative logarithm of the *p*-value [–log_10_(*p*)], derived from Fisher’s exact test, which compares the frequency of each GO term in the input gene set to its expected frequency in the complete *A. thaliana* genome annotation. Thus, a higher score indicates stronger statistical support for the overrepresentation of a given functional category.

### 4.5. Pathway-Level Annotation Using the Plant Reactome Database

To investigate the biological context and potential regulatory roles of the identified MRTFs, pathway-level annotation was performed using the Plant Reactome pathway database, integrated within the OmicsBox functional analysis environment (BioBam Bioinformatics, Valencia, Spain). This analysis was conducted by mapping the MRTFs to curated biological pathways based on their corresponding gene identifiers in *A. thaliana*.

The Plant Reactome database provides a high-confidence, manually curated framework of plant-specific pathways, including hormonal signaling, metabolic routes, developmental transitions, and environmental response mechanisms. Within OmicsBox, the pathway annotation module assigns genes to Plant Reactome pathways using cross-referenced UniProt and Ensemble identifiers, and links them to specific biological processes based on existing literature and experimental data [73].

Functional assignment was based on exact or high-confidence orthologous relationships curated in the Reactome database. Thus, this analysis highlights known and putative roles of MRTFs in pathway networks, supporting their integration into developmental and environmental regulatory circuits.

### 4.6. Gene Regulatory Network Construction and Visualization

A floral MADS-box transcription factor regulatory network was constructed to visualize and analyze key regulatory interactions during flower development. The network topology was based on experimentally validated regulatory relationships, primarily derived from the literature [1,74]. Genes were represented as nodes, and directed edges indicated transcriptional regulation between them. Special emphasis was placed on genes with known autoregulatory feedback, such as *PI* and *SEP3*.

Network construction and topological analysis were performed using the NetworkX (version 3.5) Python library. Key topological metrics—including in-degree, out-degree, and clustering coefficient—were calculated for each node to assess their regulatory roles within the network. These metrics provided insight into the connectivity and potential regulatory influence of each gene.

The network was visualized using Matplotlib (v3.6.0 or later) in Python with node coloring based on functional criteria. Genes exhibiting autoregulation or peak expression levels (as determined by RNA-seq data) were highlighted in magenta to denote their potential role as central regulators.

### 4.7. Predicted DNA-Binding Interactions of PI and SEP3 Proteins

The amino acid sequences of *A. thaliana* PISTILLATA (UniProt ID: P48007, 208 aa; sequence:

MGRGKIEIKRIENANNRVVTFSKRRNGLVKKAKEITVLCDAKVALIIFASNGKMIDYCCPSMDLGAMLDQYQKLSGKKLWDAKHENLSNEIDRIKKENDSLQLELRHLKGEDIQSLNLKNLMAVEHAIEHGLDKVRDHQMEILISKRRNEKMMAEEQRQLTFQLQQQEMAIASNARGMMMRDHDGQFGYRVQPIQPNLQEKIMSLVID) and SEPALLATA3 (UniProt ID: Q9LFS3, 251 aa) were obtained from UniProt. Promoter regions were retrieved from the EPDs: *PI* (AT5G20240) at –1040 to –1000 (CACTGCTCCTTTTTCTTTTTTTTTTTTCCTTTTTCTAAACC) and –3870 to –3830 (AGGTTTCGATTTGATTTCCTTTTGTGGTGACAGTTCCATGT); *SEP3* (AT1G24260) at +446 to +496 (TGTTTCTCTTTGATTTCCATTTTTGTTTTTGATTTTTTTTCTATTTCTCTT).

Three-dimensional protein structures were predicted using AlphaFold3 https://alphafoldserver.com/ (accessed on 1 November 2025) in both monomer and multimer modes with default parameters. Protein–DNA complex models were generated with the AlphaFold3 protein–nucleic acid module using the above promoter fragments as ligands.

### 4.8. Gene Expression Analysis

To evaluate relative expression levels of MADS-box genes and relevant TFs in *A. thaliana* floral tissue, raw RNA-seq data corresponding to ERR6824158 (library size: 8754754), ERR6824159 (library size: 8305920), and ERR6824160 (library size: 8061550) were downloaded from the NCBI Sequence Read Archive accessed on 01.08.2025. These samples were derived from wild-type flower tissue of *A. thaliana* described earlier [75]. RNA-seq paired-end reads were first quality trimmed with Trimmomatic v0.39 [76].Cleaned reads were aligned to the *A. thaliana* TAIR10 reference using the Bowtie2 [77], plugin in Geneious Prime (version 2025.0), and gene expression levels were quantified as RPKM values using the built-in Geneious Prime calculation method. For each sample, reads were mapped directly to exon-transcript models of the investigated genes, facilitating targeted expression quantification. Gene expression was quantified using RPKM normalization (Equation (1)), which corrects for both gene length and sequencing depth. RPKM values were calculated for each gene in each of the three floral replicates.

(1) $$RPKM=\frac{{C\times 10}^{9}}{N\times L}$$

**Equation (1).** RPKM, where *C* is the number of reads mapped to the gene, N is the total number of mapped reads in the sample, and L is the gene length in base pairs.

### 4.9. PWM-Based TFBS Identification and p-Value Calculation

We utilized binding site predictions as reported by the JASPAR and EPD database tools (accessed on 1 March 2025), based on PWM scanning. In the case of JASPAR, each candidate site is assigned a score. The weight score is calculated as the logarithm of the likelihood ratio (Equation (2)).

(2) $$Weight=log\left[\frac{P\left(Site|Matrix\right)}{P\left(Site|Background\right)}\right]$$

**Equation (2).** Weight score, where the numerator represents the likelihood under the PWM and the denominator represents the likelihood under a nucleotide background model.

From this score, a *p*-value is derived, reflecting the probability of observing such a match by chance under the background model [18].

The EPD offers curated promoter PWMs and allows scanning for motif hits, though its documentation primarily focuses on promoter sequence extraction and motif mapping rather than on detailed statistical modeling.

### 4.10. Expression Analysis of MRTFs After BABA-Treatment

Cultivation of plant materials, treatment and Illumina sequencing were performed as described by Virág et al., 2024 [21]. In brief, A. thaliana and Hordeum vulgare plants were grown under controlled environmental conditions and treated with β-aminobutyric acid (BABA, 250 µM) to induce resistance responses. Leaf tissues from both BABA-treated and control plants were collected for RNA extraction. Libraries were sequenced using a single-end option, and the final output consisted of 14–26 M × 85 base pairs’ (bp) long reads (1.19–2.21 Gbp).

In this experiment fresh leaves of 17-day-old plant samples were collected (72 h after the BABA treatment) for NGS sequencing in three biological replications [21]. Samples were stored in DNA/RNA Shield (Zymo research, Irvine, CA, USA) at −25 °C until sequencing. For in silico analysis, transcript abundances were determined using RNA sequencing using Geneious 2025.0 (as described above). Gene expression levels were normalized as Reads Per Million (RPM) to allow direct comparison between samples with different sequencing depths. Differential expression analysis was performed by comparing RPM values between BABA-treated and control groups to identify transcriptional changes associated with BABA-induced regulatory responses. The transcript abundance matrix was deposited in Mendeley database https://doi.org/10.17632/nzyw9csjs8.3, file name: AT-BABA-transcript-abundancies (accessed on 1 September 2025).

## 5. Conclusions

This study presents a systematic in silico analysis of TFBS distributions across 18 core flowering genes in *A. thaliana*. The identification of candidate MRTFs, together with the delineation of gene-specific versus shared TFBS profiles, underscores the hierarchical and combinatorial nature of transcriptional control during floral development. These results are significant, because they establish a promoter-level framework for understanding how diverse regulatory pathways converge on key flowering regulators. The compiled TFBS dataset provides a foundation for future research, including experimental validation of autoregulatory and cross-regulatory modules, the development of predictive network models, and comparative studies across plant species, hereby opening perspectives for applicational advances in crop flowering regulation.

## Figures and Tables

**Table 1:** Summary of 18 core flowering-regulatory genes in *A. thaliana*, whose promoter motifs were analyzed.

Gene (Symbol)	TAIR ID	Protein Family	Biological Processes (GO:BP)	Molecular Function (GO:MF)	Cellular Component (GO:CC)
*SEP3*	AT1G24260	MADS-box	Floral organ identity, ovule and seed coat development	DNA-binding TF, dimerization	Nucleus
*FT*	AT1G65480	PEBP (Florigen)	Flower development, photoperiodism, meristem determinacy	Lipid binding, protein binding	Nucleus, ER
*AP1*	AT1G69120	MADS-box	Floral meristem determinacy, meristem structure	DNA-binding TF, heterodimerization	Nucleus
*SEP4*	AT2G03710	MADS-box	Floral meristem identity, organ development	DNA-binding TF, dimerization	Nucleus
*SVP*	AT2G22540	MADS-box	Repression of flowering, temperature response	TF activity, translational repression	Nucleus
*SOC1* (*AGL20*)	AT2G45660	MADS-box	Flowering, gibberellin response, meristem identity	DNA-binding TF, dimerization	Nucleus, cytoplasma
*SEP2*	AT3G02310	MADS-box	Ovule development, cell differentiation	DNA-binding TF, dimerization	Nucleus
*AP3*	AT3G54340	MADS-box	Floral organ identity	DNA-binding TF, dimerization	Nucleus
*AG*	AT4G18960	MADS-box	Flower development	DNA-binding TF, dimerization	Nucleus
*AGL24*	AT4G24540	MADS-box	Vernalization response, flowering transition	DNA-binding TF, homo- and heterodimerization	Nucleus, cytoplasma
*FD*	AT4G35900	bZIP	Flower development, photoperiodism	DNA-binding TF, protein binding	Nucleus
*AP2*	AT4G36920	AP2/ERF	Floral organ identity, meristem maintenance	TF activity, *cis*-regulatory binding	Nucleus
*TFL1*	ATS5G03840	PEBP	Repression of flowering, meristem determinacy	Co-regulatory activity	Nucleus, vacuole, plasma membrane
*FLC*	ATS5G10140	MADS-box	Vernalization response, circadian rhythm, flowering repression	DNA-binding TF, dimerization	Nucleus protein complex
*SEP1*	ATS5G15800	MADS-box	Ovule development, transcription regulation	DNA-binding TF, dimerization	Nucleus
*PI*	AT5G20240	MADS-box	Floral organ identity	DNA-binding TF, dimerization	Nucleus, cytoplasma
*FUL*	ATS5G60910	MADS-box	Flower/fruit development, morphogenesis	DNA-binding TF, dimerization	Nucleus
*LFY*	ATS5G61850	LFY-specific	Floral meristem determinacy, GA signaling	DNA-binding TF, chromatin binding, dimerization	Nucleus

**Table 2:** The number of predicted binding TFs in the 18 investigated upstream and downstream promoter regions in *A. thaliana*. Values are shown for two significance thresholds (*p* < 0.0001 and *p* < 0.00001). The number of TFBSs was investigated in the promoter regions within the range of −5000 to +1000 relative to the TSS.

Gene Name	EPD/TAIR ID	Number of TF Binding Positions (*p* < 0.0001)		Number of TF Binding Positions (*p* < 0.00001)	
		Upstream	Downstream	Upstream	Downstream
*SEPALLATA* (*SEP3*), *AGL9*	AT1G24260_1	788	126	181	39
*FLOWERING LOCUS T* (*FT*)	AT1G65480_1	617	201	136	40
*APETALA* (*AP1*)	AT1G69120_1	707	159	159	24
*SEPALLATA (SEP4*), *AGL3*	AT2G03710_1	720	210	176	64
*SHORT VEGETATIVE PHASE* (*SVP*)	AT2G22540_1	719	133	185	24
*SUPPRESSOR OF CONSTANS OVEREXPRESSION1* (*SOC1*), *AGL20*	AT2G45660_1	710	156	137	56
*SEPALLATA* (*SEP2*), *AGL4*	AT3G02310_1	695	121	152	27
*APETALA3* (*AP3*)	AT3G54340_1	638	126	146	20
*AGAMOUS* (*AG*)	AT4G18960_1	624	137	154	37
*AGL24*	AT4G24540_1	563	147	76	34
*FLOWERING LOCUS D* (*FD*)	AT4G35900_1	716	163	156	38
*APETALA* (*AP2*)	AT4G36920_1	752	188	202	51
*TERMINAL FLOWER* (*TFL1*)	AT5G03840_1	636	131	155	15
*FLOWERING LOCUS C* (*FLC*), *AGL25*, *FLF*	AT5G10140_1	687	178	117	45
*SEPALLATA* (*SEP1*), *AGL2*	AT5G15800_1	592	109	94	25
*PISTILLATA* (*PI*)	AT5G20240_1	855	87	180	21
*FRUITFULL*, *AGL8*, (*FUL*)	AT5G60910_1	680	198	144	64
*LEAFY* (*LFY*)	AT5G61850_1	502	345	99	105

## Data Availability

The dataset has been deposited to Mendeley Data (https://doi.org/10.17632/nzyw9csjs8.2, accessed on 1 June 2025) under the title “Collection of TF-binding sites of 18 central flowering genes predicted by PWM in the *A. thaliana* genome”. It consists of two folders, each containing 18 text files (2 × 18), corresponding to different stringency thresholds applied during the prediction of TFBS. Each file contains detailed information on the predicted binding positions of *A. thaliana* TFs in the upstream and downstream regions of the coding sequences of the analyzed genes. TFBS positions are provided in the files relative to the TSS. The filenames are structured to convey essential metadata, including species name, gene identifier, and the applied stringency threshold (e.g., *A. thaliana*-AT1G24260_1-0.00001.txt). The files, which list PWM hits identified at specific significance levels, are organized into directories according to the corresponding *p*-value threshold used during analysis. For each TF, binding site counts are reported separately for the upstream and downstream regions. These data are categorized by gene and by threshold, enabling direct comparison of TF-binding patterns across different promoter segments and significance levels. The RNA-seq data were obtained from the NCBI SRA (accessions ERR6824158, ERR6824159, and ERR6824160), corresponding to *A. thaliana* flower samples.

## Supplementary Materials

The following supporting information can be downloaded at https://www.mdpi.com/article/10.3390/ijms262211152/s1.
