# Supplementary material for: Promoter Motif Profiling and Binding Site Distribution Analysis of Transcription Factors Predict Auto- and Cross-Regulatory Mechanisms in Arabidopsis Flowering Genes
Source: Int J Mol Sci. 2025 Nov 18;26(22):11152. doi: 10.3390/ijms262211152 (PMC12652660; doi:10.3390/ijms262211152)
Supplement: Supplementary file 1 [file ijms-26-11152-s001.zip › Supplementary Table S1.pdf]

Table S1. Predicted binding sites of the core MRTFs BPC1 and OBP3 within the proximal promoter regions (−500 to +200 bp) of 18 flowering regulatory genes in *A. thaliana* ($p < 0.0001$).

Gene	BPC1_sites	OBP3_sites
SEP3	29, -6, -62	25, -90
FT	-166	-424, -172
AP1	-401	8, -373
SEP4	-298, -258	-417, -297, -188
SVP	-280, -48, 2, 53	-469, -272, -228, 58
SOC1	110, -14	147, 25, -8, -246
SEP2	112, -58, -118	131, 85, 35
AP3	69, 25, -27, -57	-319
AG	-50	-54
AGL24	17	-338, -413, -453
FD	-284, -227, -35, 57	-429
AP2	-220, -124, 102	-346, -179, -130, -102, 55
TFL1	16, -64, -132	-67, -136
FLC	18	20
SEP1	-53, -80	107, -59
PI	-87, 39	-278, -249, -223
FUL	94, 20, -172	-50, -174
LFY	-88, 40	-339, 38
