# Supplementary material for: Promoter Motif Profiling and Binding Site Distribution Analysis of Transcription Factors Predict Auto- and Cross-Regulatory Mechanisms in Arabidopsis Flowering Genes
Source: Int J Mol Sci. 2025 Nov 18;26(22):11152. doi: 10.3390/ijms262211152 (PMC12652660; doi:10.3390/ijms262211152)
Supplement: Supplementary file 1 [file ijms-26-11152-s001.zip › Supplementary Figure S2.pdf]

Figure S2

Python script to filter values from the dataset to identify differences in upstream (-) and downstream (+) TF-binding records.

```
from os import scandir

# Path and names:
folder1 = "C:\\Data\\0.0001\\"
folder2 = "C:\\Data\\0.00001\\"
name_ID_file = "C:\\Data\\Name_ID.txt"
result_file = "C:\\Data\\Result_Up_Down.txt"

# Containers:
container = {}
ID_genename = {}

# Load data to "container" from files:
dirlist = scandir(folder1)
for file in dirlist:
    ID = file.name[12:len(file.name)-11]

    upstr000 = 0
    downstr000 = 0
    upstr0000 = 0
    downstr0000 = 0

    reader1 = open(folder1 + file.name)
    reader2 = open(folder2 + file.name.replace("1.txt","01.txt"))

    reader1.readline()
    reader2.readline()

    row1 = reader1.readline().removesuffix("\n")
    row2 = reader2.readline().removesuffix("\n")

    while True:
        if not row1:
            break
        fields1 = row1.split("\t")
        if not fields1[1] == "no hit found.":
            upstr000 += sum(1 for x in fields1[1:] if int(x) < 0)
            downstr000 += sum(1 for x in fields1[1:] if int(x) > 0)
        fields2 = row2.split("\t")
        if not fields2[1] == "no hit found.":
            upstr0000 += sum(1 for x in fields2[1:] if int(x) < 0)
            downstr0000 += sum(1 for x in fields2[1:] if int(x) > 0)
        row1 = reader1.readline().removesuffix("\n")
        row2 = reader2.readline().removesuffix("\n")
    reader1.close()
    reader2.close()
    container[ID] = (upstr000,downstr000,upstr0000,downstr0000)
```

```
# Load ID-name pairs to "ID_genename" from "name_ID_file" file:
reader = open(name_ID_file,"tr")
row = reader.readline().removesuffix("\n")
while True:
    if not row:
        break
    fields = row.split("\t")
    ID_genename[fields[1]] = fields[0]
    row = reader.readline().removesuffix("\n")
reader.close()

# Write result table:
writer = open(result_file,"tw")
for key in container:
    writer.write(ID_genename[key] + "\t")
    writer.write(key + "\t")
    writer.write(str(container[key][0]) + "\t")
    writer.write(str(container[key][1]) + "\t")
    writer.write(str(container[key][2]) + "\t")
    writer.write(str(container[key][3]) + "\n")
writer.close()
```
