# Supplementary material for: Promoter Motif Profiling and Binding Site Distribution Analysis of Transcription Factors Predict Auto- and Cross-Regulatory Mechanisms in Arabidopsis Flowering Genes
Source: Int J Mol Sci. 2025 Nov 18;26(22):11152. doi: 10.3390/ijms262211152 (PMC12652660; doi:10.3390/ijms262211152)
Supplement: Supplementary file 1 [file ijms-26-11152-s001.zip › Supplementary Figure S1.pdf]

Supplementary Figure S1

The figure illustrates the autopromoter and promoter-binding interactions of the PISTILLATA and SEPALLATA3 transcription factors as predicted by AlphaFold3.

A

Predicted interactions between PISTILLATA amino acid residues and its promoter in –3870 to –3830 region

Amino acid (PI)	Functional group	Number of hydrogen bonds	Promoter nucleotide	Binding site	Type of interaction
ARG3	NH group	1	DG27	Base	Hydrogen bonds
LYS23	NH_3 group	1	DG25	Base	Hydrogen bond
LYS23	NH_3 group	2	DG26	Base	Hydrogen bond
LYS23	NH_3 group	1	DG27	Base	Hydrogen bond
ARG24	NH_3 group	3	DG26	Phosphate group	Hydrogen bonds
LYS30	NH_3 group	2	DT13	Phosphate group	Hydrogen bonds

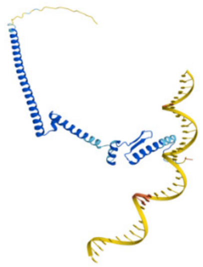

PI-PI interaction

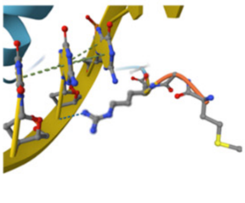

ARG3

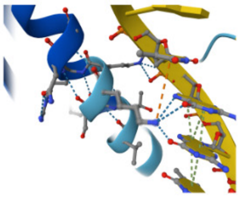

LYS23

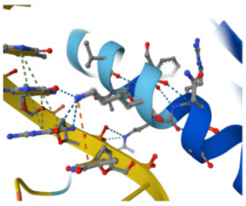

LYS23

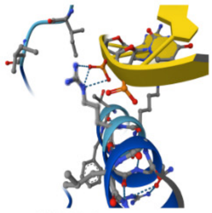

ARG24

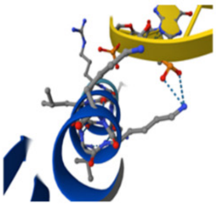

LYS30

B

Predicted interactions of PI-SEP3 DIMER amino acid residues with the PI promoter in –3870 to –3830 region

Amino acid (Protein)	Functional group	Number of hydrogen / covalent bonds	Promoter nucleotide	Binding site	Type of interaction
LYS23 (PI)	NH_3 group	1 hydrogen bond	DG27	Base	Hydrogen bond
ARG24 (PI)	NH_3 group	2 hydrogen bonds	DG26	Phosphate group	Hydrogen bonds
ARG3 (PI)	NH group	2 hydrogen bonds	DG27	Base	Hydrogen bonds
ARG3 (SEP3)	NH group	Multiple covalent + hydrogen bonds	DT20	Base	Covalent and H-bonds

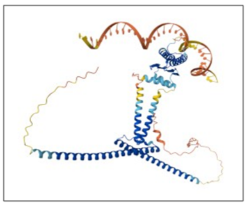

PI-SEP3 DIMER

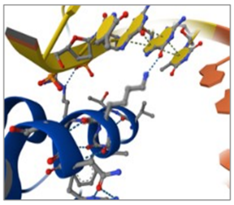

LYS23 (PI)

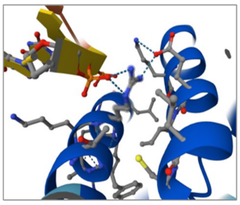

ARG24 (PI)

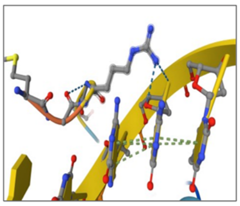

ARG3 (PI)

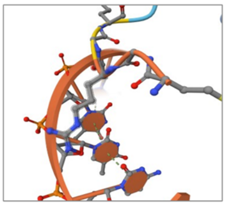

ARG3 (SEP3)

C

Predicted interactions between the PI–SEP3 DIMER and the PISTILLATA promoter in–1040 to –1000 region

Amino acid (Protein)	Functional group	Number of hydrogen / covalent bonds	Promoter nucleotide	Binding site	Type of interaction	Comment
ARG3 (SEP3)	NH group	Multiple covalent bonds	DT18, DT19	Base	Covalent interactions	Direct binding observed in model

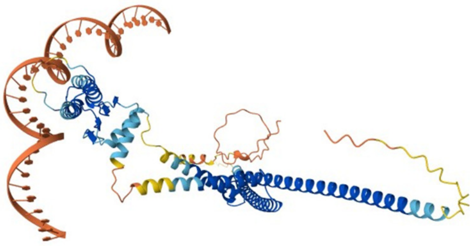

PI–SEP3 DIMER

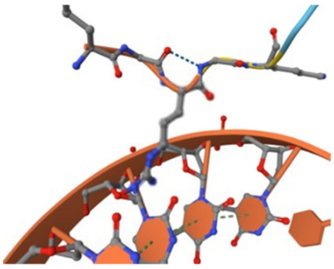

ARG3 (SEP3)

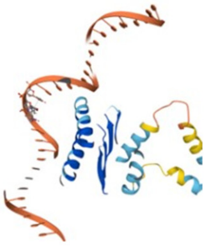

PI–SEP3 DIMER

D

Predicted interactions between the PI–SEP3 DIMER and the SEPALLATA3 promoter in +446 to +496 region

Amino acid (Protein)	Functional group	Number of hydrogen bonds	Promoter nucleotide	Binding site	Partner molecule	Type of interaction
ARG24 (PI)	NH group	1	DG31	Phosphate group	SEP3 promoter + SEP3 GLU34	Hydrogen bond (DNA + protein)
LYS30 (PI)	NH_3 group	1	DT29	Phosphate group	SEP3 promoter	Hydrogen bond

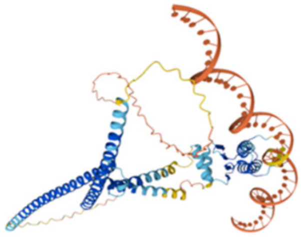

PI–SEP3 DIMER

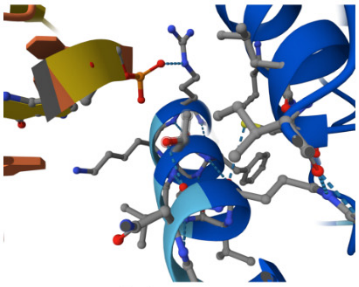

ARG24 (PI)

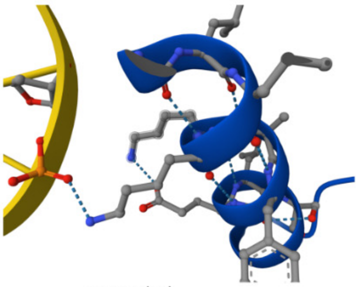

LYS30 (PI)
